# Supplementary material for: Efficient production of itaconic acid from the single-carbon substrate methanol with engineered Komagataella phaffii
Source: Biotechnol Biofuels Bioprod. 2024 Jul 15;17:98. doi: 10.1186/s13068-024-02541-1 (PMC11251334; doi:10.1186/s13068-024-02541-1)
Supplement: Supplementary file 1 — Supplementary Material 1. [file 13068_2024_2541_MOESM1_ESM.docx]

**Efficient production of itaconic acid from the single carbon substrate methanol with engineered *Komagataella phaffii***

Manja Mølgaard Severinsen^1^, Simone Bachleitner^1^, Viola Modenese^1,3^, Özge Ata^1,2^, Diethard Mattanovich^1,2^

^1^ BOKU University, Vienna, Department of Biotechnology, Institute of Microbiology and Microbial Biotechnology, 1190 Vienna, Austria

^2^ Austrian Centre of Industrial Biotechnology, Vienna, 1190, Austria

^3^ Department of Food, Environmental and Nutritional Sciences, University of Milan, Milan, Italy

* Corresponding author: diethard.mattanovich@boku.ac.at

**Table S1**. **Primer names and sequences.**

| **Name** | **Sequence** | **Comments** |
| --- | --- | --- |
| **Primers used for diagnostic verification of generated strains** | | |
| dia_GUT1_check_fwd | GATTCCAAACTGCAGGAACGCAG | Primers bind outside the *GUT1* locus and are used for verification of *GUT1* insertions. |
| dia_GUT1_check_rev | GAGGAGTCGGCAAAGTACCC |  |
| dia_RGI_check_fwd | ATCAAACTTTTTGAATGGA | Primers bind outside the *RGI2* locus and are used for verification of *RGI2*-directed inserts. |
| dia_RGI_check_rev | CTATAAGAAACTGGAGACT |  |
| BB2_check_fwd | CTGCGTTATCCCCTGATTCT | Primers used to verify integration of transcription unit in BB2 plasmids. |
| BB2_check_rev | GGGTGAGCAAAAACAGGAAG |  |
| BB3_check_fwd | TTAGTATGCTGTGCTTGGGTG | Primers used to verify multicopy integration of the heterologous genes. The respective genes were told apart based on size observed via gel electrophoresis. |
| BB3_check_rev | GAGGTATGTAGGCGGTGCTA |  |
| **Primers used for RT-qPCR analysis** | | |
| q_act1_fw | CCTGAGGCTTTGTTCCACCCATCT | Primers used to analyze gene expression via RT-qPCR |
| q_act1_rv | GGAACATAGTAGTACCACCGGACATAACGA |  |
| q_cadA_fw | GGGTCGCGTGAGGATTGAGTTC |  |
| q_cadA_rv | CTACCCGCAAGGGTTCGGTAT |  |
| q_mttA_fw | GCAACACCTTCAACTGCGTCAA |  |
| q_mttA_rv | GACCTTCTCGTAAACGGGGAACAT |  |
| q_mfsA_fw | AGTGCGTCATCACCTTCGTC |  |
| q_mfsA_rv | AGGGAGGAGTAGCCCATAGC |  |

Primers used for diagnostic verification of the generated strains, for RT-qPCR analysis and for cDNA synthesis.

**Table S2. Process parameters of the respective fed-batch cultivations.**

| Total cultivation time [h] | MeOH phase time [h] | Strain | Temperature | Initial batch volume [mL] (Bioreactor model) | MeOH flow rate  [g·L^-1^·h^-1^] | Feed-medium flow rate [mL·h^-1^] | Volume [mL] | YDM [g·L^-1^] |
| --- | --- | --- | --- | --- | --- | --- | --- | --- |
| 126 | 73 | cadA+mttA | 25 °C | 320 (SR0700ODLS) | 0.028·t+0.6 | 0.225·t+1.95  3.65-0.111·t | 556.7 | 140.2 |
|  |  | cadA+mttA+mfsA_pGAP_ |  |  |  |  | 552.2 | 138.8 |
| 65 | 40 | cadA+mttA+mfsA_pGAP_ | 25 °C | 820 (SR1000ODLS) | 3.1 | 10.3 | 1028.5 | 76.1 |
|  |  |  | 30 °C |  | 3.1 |  | 1053.3 | 66.3 |
| 138 | 114 | cadA+mttA+mfsA_pGAP_ | 30 °C | 320 (SR0700ODLS) | 2.0 | 4 | 447.2 | 66.6 |
|  |  | MC I |  |  | 2.0 |  | 476.1 | 62.6 |
|  |  | MCII |  |  | 1.9 |  | 470.7 | 67.0 |
|  |  | MCIII |  |  | 2.5 |  | 449.1 | 48.0 |
| 137 | 115 | MC I | 28 °C | 320 (SR0700ODLS) | 1.6 | 4 | 464.5 | 65.8 |
|  |  |  | 30 °C |  | 1.8 |  | 472.3 | 63.7 |
|  |  |  | 32 °C |  | 2.2 |  | 451.9 | 65.9 |
|  |  |  | 34 °C |  | 1.8 |  | 443.2 | 60.6 |

Total cultivation time [h], duration of MeOH phase [h], strain, temperature, batch volume and reactor model, average MeOH flow rate [g·L^-1^·h^-1^], glycerol-feed flow rate [mL·h^-1^] and final volume [mL], final itaconic acid titer [g·L^-1^] and yeast dry cell mass (YDM) [g·L^-1^] are given.


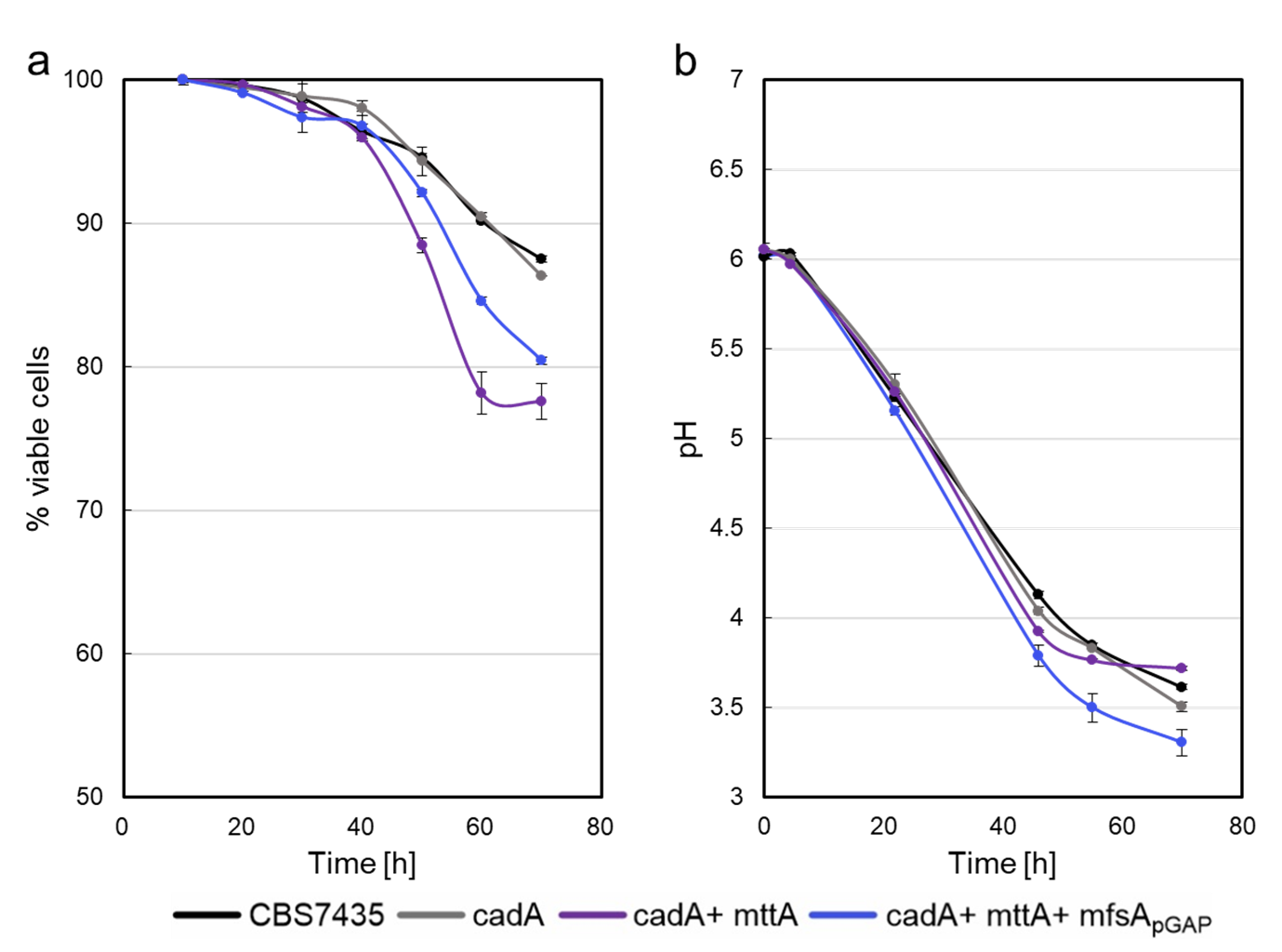


**Figure S1. Viability of strains during pH change.** During the comparative shake flask cultivation of the *K. phaffii* strain CBS7435 with the generated strains (cadA, cadA+mttA and cadA+mttA+mfsA_pGAP_ **a)** viability of the strains was estimated with PI-staining at every sampling point, whilst **b)** pH was also measured. The strains were cultivated in biological duplicates and averages and standard deviations are shown for both viability and pH.


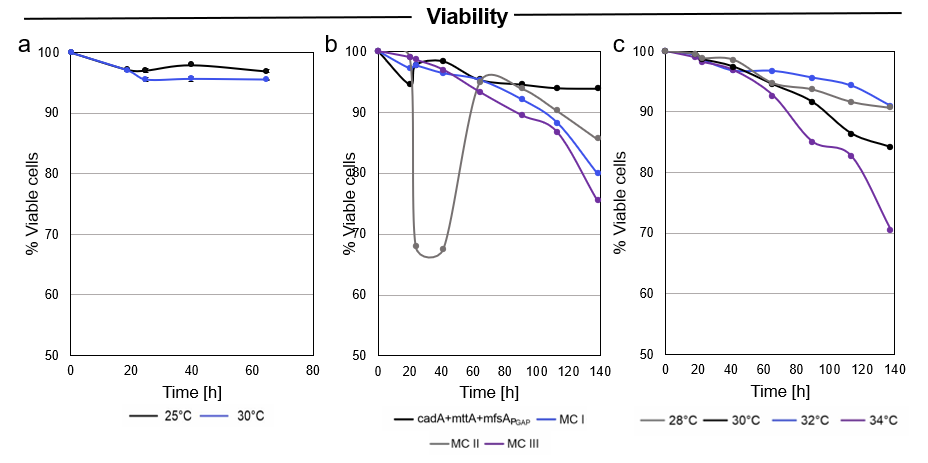


**Figure S2. Viability of strains in fed-batch cultivations. a)** The viability of the cells remained high during the fed-batch cultivation where an increased cultivation temperature of 30 °C was compared to 25 °C. In **b)** the viability of the multicopy strains (MC I, MC II and MC III) during the fed-batch cultivation at 30 °C is shown. Whilst the cadA+mttA+mfsA_pGAP_ and the MC I, MC III have a stable decrease in viability over time, the MC II shows a decreased viability (> 67.5 %) at the end of the batch phase. Upon methanol induction, the viability did however recover during the methanol feed phase. In **c)** the viability of the MC I strain when cultivated at 28, 30, 32 and 34 °C is shown, at 34 °C the greatest drop in viability (70.5 %) over time is experienced.


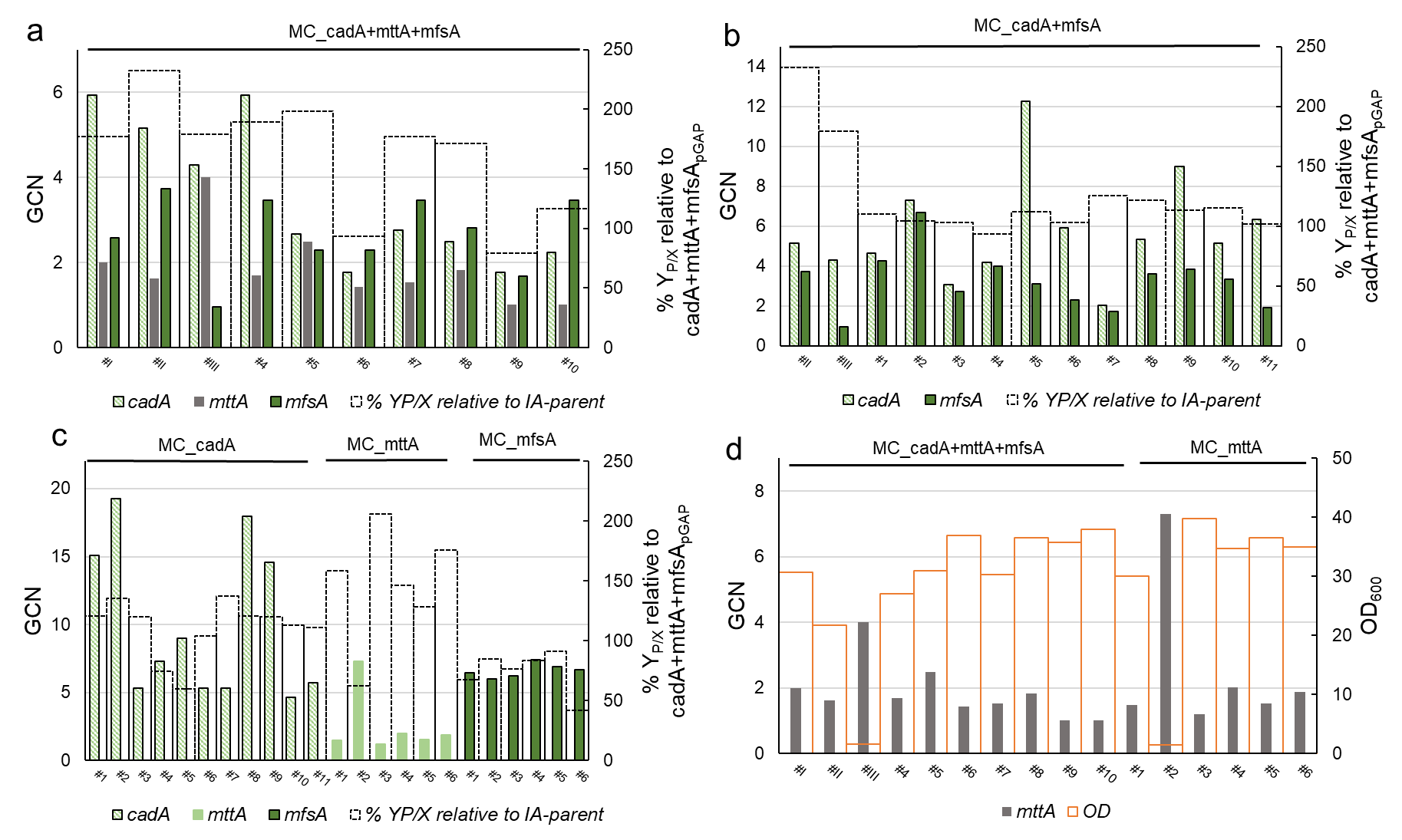


**Figure S3. The gene copy numbers (GCN) of the three heterologous genes (*cadA*, *mttA*, *mfsA*) of the generated multicopy strains (MC) was investigated via RT-qPCR relative to the cadA+mttA+mfsA_pGAP_ strain**. In **a-c)** GCN of multicopy strains is shown with the relative yield obtained in the 24-deep-well plate screening in comparison to the cadA+mttA+mfsA_pGAP_ strain. In **d)** the GCN of *mttA* in the MC_mttA and MC_cadA+mttA+mfsA is shown with final OD of the 24-deep well plate screening.

**Table S3. OD_600_ and itaconic acid obtained from 24-deeep-well plate screening after 48 hours.**

| Strain | Clone no. | OD_600_ | Itaconic acid [g·L^-1^] |
| --- | --- | --- | --- |
| cadA+mttA+mfsA_pGAP_ |  | 31.4 | 1.5 |
| MC_cadA+mttA+mfsA | #I | 30.8 | 2.8 |
|  | #II | 21.8 | 2.6 |
|  | #III | 1.6 | 0.1 |
|  | #4 | 27.0 | 2.6 |
|  | #5 | 31.0 | 3.2 |
|  | #6 | 37.0 | 1.8 |
|  | #7 | 30.3 | 2.7 |
|  | #8 | 36.5 | 3.2 |
|  | #9 | 35.8 | 1.5 |
|  | #10 | 38.0 | 2.3 |
| MC_cadA+mfsA | #1 | 34.5 | 1.9 |
|  | #2 | 34.5 | 1.8 |
|  | #3 | 37.8 | 2.0 |
|  | #4 | 37.5 | 1.8 |
|  | #5 | 32.8 | 1.9 |
|  | #6 | 36.8 | 1.9 |
|  | #7 | 39.0 | 2.5 |
|  | #8 | 30.3 | 1.9 |
|  | #9 | 35.8 | 2.1 |
|  | #10 | 37.5 | 2.2 |
|  | #11 | 36.5 | 1.9 |
| MC_cadA | #1 | 30.8 | 1.9 |
|  | #2 | 27.0 | 1.9 |
|  | #3 | 38.8 | 2.4 |
|  | #4 | 24.5 | 0.9 |
|  | #5 | 6.2 | 0.2 |
|  | #6 | 36.3 | 1.9 |
|  | #7 | 32.5 | 2.3 |
|  | #8 | 38.8 | 2.4 |
|  | #9 | 37.0 | 2.3 |
|  | #10 | 36.3 | 2.1 |
|  | #11 | 39.8 | 2.3 |
| MC_mttA | #1 | 30.0 | 2.4 |
|  | #2 | 1.4 | 0.0 |
|  | #3 | 39.8 | 4.2 |
|  | #4 | 34.8 | 2.6 |
|  | #5 | 36.5 | 2.4 |
|  | #6 | 35.0 | 3.2 |
| MC_mfsA | #1 | 36.5 | 1.3 |
|  | #2 | 36.8 | 1.6 |
|  | #3 | 41.5 | 1.6 |
|  | #4 | 42.8 | 1.8 |
|  | #5 | 44.8 | 2.1 |
|  | #6 | 28.5 | 0.6 |

Strain name and clone number, OD_600_ and itaconic acid titer [g·L^-1^] measured after 48 hours of cultivation in 24-deep-well plate are given.


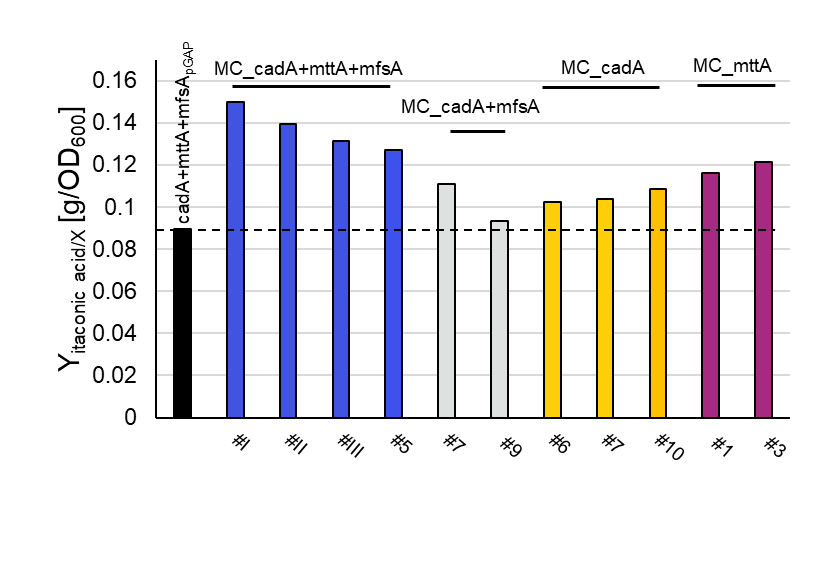


**Figure S4. Shake flask screening with multicopy strains.** A set of multicopy clones with different genotypes were selected for further investigation in a shake flask screening with the parent strain, cadA+mttA+mfsA_pGAP_, as reference. The itaconic acid yields per biomass after 70 hours are shown.


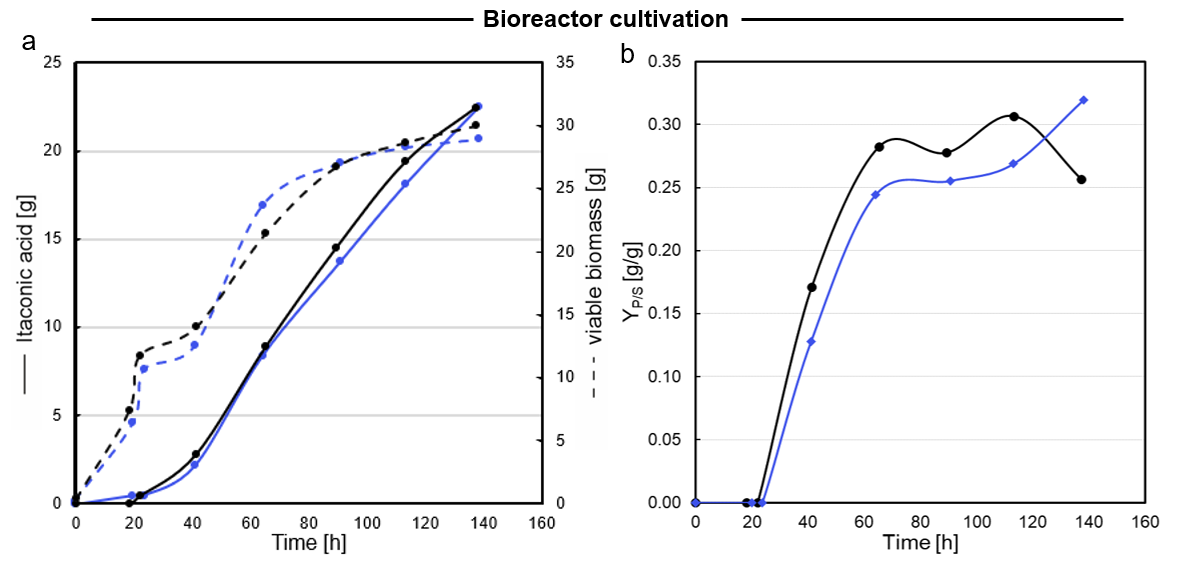


**Figure S5. Overlay of fed-batch cultivations performed at 30°C with MC I.** In blue is the fed-batch cultivation of MC I cultivated at 30°C displayed in Figure 6, in black is the fed-batch cultivation of MC I cultivated at 30°C displayed in Figure 8. **a)** Growth and production profiles are shown for the both fed-batch cultivations performed with MC I cultivated at 30°C. **b)** the yield of itaconic acid per gram of methanol during the fed-batch cultivation.
